# Selecting sires to improve reproductive success: key traits for enhanced fertility and embryo development

**DOI:** 10.1590/1984-3143-AR2025-0052

**Published:** 2025-08-14

**Authors:** Froylan Sosa, Martha Sofia Ortega

**Affiliations:** 1 Department of Animal and Dairy Sciences, University of Wisconsin-Madison, Madison, WI, United States

**Keywords:** sire fertility, embryo development, paternal contributions

## Abstract

Embryo development is a complex process that requires several physiological and molecular events to happen harmoniously, and all of this begins with the interaction of the oocyte and sperm. The ability of an oocyte to become a healthy blastocyst is the result of several critical events that are determinants for the successful development of the embryo. Among these events are the sperm's ability to interact with and penetrate the oocyte, carry out syngamy, the developmental competence of the oocyte to support mitotic divisions, and the proper activation of the molecular machinery to regulate the embryo's developmental competence during the early stages of embryonic development. Some of these events originate from either the paternal or maternal side. The focus of this review is to explore the contributions of the paternal side to reproduction in general, with greater emphasis on early embryo development. A deeper understanding of these paternal factors and their influence on embryo development and overall fertility will support the development of new strategies for selecting sires to improve reproductive efficiency in cattle.

## Introduction

A considerable amount of research has focused on understanding how maternal contributions regulate embryo development in several mammalian species, primarily by examining how the maternal environment affects embryo development and impacts pregnancy success (Moraes and Hansen, 1997; Tríbulo et al., 2018; Jeensuk et al., 2022; Sosa and Hansen, 2023; Sosa et al., 2023). The study of paternal contributions to embryo development is promising as it may provide new insights into the physiological and molecular mechanisms of embryo development. Reports have shown that the sire plays a critical role in reproductive success, influencing not only fertilization but also blastocyst formation, and pregnancy rates (Kidder et al., 1954; Bearden et al., 1956; Ward et al., 2001; Al Naib et al., 2011; Kumaresan et al., 2017; Bernecic et al., 2021; Hidalgo et al., 2021; O’Callaghan et al., 2021; Fernández-Montoro et al., 2025). A summary of the consequences of different phenotypes affecting sperm function on embryo development is presented in Table 1. However, the molecular and physiological mechanisms by which the sire can affect fertility are less understood. The goal of this review is to describe potential paternal factors that regulate events before and after fertilization, as well as during the early stages of embryonic development. Understanding the regulation of these critical events will provide valuable insights for developing strategies to select sires for enhanced fertility.

**Table 1:** Comparison of embryo development across different sperm phenotypes and treatments.

**Phenotype/** **treatment**	**Item**						
	**Fertilization 1**	**Cleavage rate 2**	**Morulae/** **cleaved 3**	**Blastocyst/** **zygotes 4**	**Blastocyst/** **cleaved 5**	**Blastocyst/** **morulae 6**	**Reference**
Sperm without acrosome defects	82.8^A^	93.8^A^	65.9^A^	32.1^A^		51.7^A^	Thundathil et al. (2000)
Sperm with acrosome defects	22.6^B^	22.7^B^	35.5^B^	2.7^B^		33.3^B^	
Sperm without proximal cytoplasmic droplets	68.7^A^	60.7^A^	43.8^A^		20.0^A^		Thundathil et al. (2001)
Sperm with proximal cytoplasmic droplets	8.8^B^	5.0^B^	0.0^B^		0.0^B^		
*Sperm with low levels of DNA damage		68±2.7^A^		15±1.9^A^			Fatehi et al. (2006)
*Sperm with high levels of DNA damage		46.0±6.6^B^		1.0±0.4^B^			
Protamine adequate levels		64.1±0.8^A^		30.3±0.8^A^	47.5±1.1^A^		Castro et al. (2018)
Protamine deficiency		64.7±1.8^A^		18.5±1.2^B^	27.6±1.5^B^		
Sperm with low levels of ROS		74.0±12.1^A^			21.8±7.1^A^		Bittner et al. (2018)
Sperm with high levels of ROS		56.6±17.5^B^			7.4±4.2^B^		
Sperm with low levels of ROS	77.8±3.6	76.7±2.5^A^		29.0±1.7^A^			Fallon et al. (2023)
Sperm with high levels of ROS	74.7±4.0	68.2±2.7^B^		19.9±1.9^B^			
^*^Sperm +glutathione		86.6±2.9^A^		43.1±3.8^A^			Hu et al. (2016)
*Sperm -glutathione		82.7±3.1^B^		35.2±11^B^			

A, B In each study, different superscript letters within a given row indicate significant differences (p < 0.05) between groups across columns. Data are presented either as means ± standard error of the mean (SEM) or as simple averages, depending on the study. *Indicate the experimental treatment by either inducing DNA damage or treating the sperm with the antioxidant glutathione, as appropriate. ^1^Percentage of structures that showed two pronucleus; ^2^Percentage of embryo structures that experienced at least one mitotic cell division; ^3^Percentage of cleaved embryos that developed to the morula stage; ^4^Percentage of putative zygotes that developed to the blastocyst stage; ^5^Percentage of cleaved embryos that developed to the blastocyst stage; ^6^Percentage of morulae that developed to the blastocyst stage. ROS: reactive oxygen species.

## Paternal factors affecting fertilization and embryo development

The ability of a sire’s spermatozoon to fertilize an egg and support the development of a healthy blastocyst depends on several key factors. These include normal sperm morphology, the ability to undergo the acrosome reaction, effective sperm-egg interaction and successful penetration of the zona pellucida (ZP), proper nuclear decondensation, and DNA integrity. Failure in any of these may impair the sperm's fertilization ability and compromise the embryo's developmental competence to reach the blastocyst stage.

For example, abnormal sperm morphology such as proximal droplets results in impaired sperm-oocyte interaction, reduced fertilization rates, and cleaved embryos that are unable to continue development (Thundathil et al., 2000, 2001). Likewise, knobbed acrosomes have impaired plasma membrane integrity and result in reduced capacitation and abnormal acrosome reaction patterns (Thundathil et al., 2002). Paternal factors not only compromise the fertilization process but can also negatively affect early embryonic development. For instance, paternal DNA damage did not impair sperm motility, fertilization, or embryonic cleavage, but led to a high proportion of 2–3 cell embryos experiencing developmental arrest (Fatehi et al., 2006). A more detailed overview of specific paternal factors influencing reproductive outcomes in bovines is presented below.

## Impact of sperm DNA integrity on fertility and embryo development

Sperm DNA integrity is a critical component for reproduction as it directly affects the quality of the genetic material delivered to the oocyte, which is essential for embryo development. DNA damage has been linked to reduced fertilization rates, impaired embryo development, and lower pregnancy outcomes in humans (Lewis and Aitken, 2005; Simon et al., 2014; Zheng et al., 2018; Borges et al., 2019; Li et al., 2024). In cattle, single- or double-strand DNA breaks in sperm induced by cellular stress have been associated with reduced fertilization rates in vivo (Ribas-Maynou et al., 2024). Moreover, low-performing sires (measured by their ability to produce blastocysts in vitro) exhibit a higher incidence of DNA damage prior to density-based sperm separation compared to high-performing sires (Fallon et al., 2023).

During spermatogenesis, double DNA strand breaks are required for proper homologous recombination, pairing, and crossover between homologous chromosomes, which is essential for maintaining genetic diversity (Gray and Cohen, 2016). Failure to repair DNA breaks in sperm during the early stages of spermatogenesis can result in the persistence of DNA damage in differentiated sperm cells with consequences later during embryo development (Middelkamp et al., 2020). Additionally, cells with this characteristic are more prone to apoptosis and oxidative stress (Esteves et al., 2021). In sires, a global proteomic comparison between sperm from high and low-fertility Holstein sires (quantified as the percentage deviation from the average conception rate of all sires with a minimum of 300 inseminations in the dataset) showed significant involvement in the G2/M DNA damage checkpoint regulation pathway, indicating potential DNA integrity issues (Peddinti et al., 2008).

In sperm cells, protamination is a biological process that replaces histones with protamine during spermatogenesis to compact DNA so that it fits in the sperm head and protects the DNA from damage caused by cellular stressors (Dogan et al., 2015). This process has also been shown to play an essential role in the repair mechanism of DNA damage caused by double-strand breaks (Cho et al., 2003). In bovine sperm, increased DNA damage has been associated with low protamine content (Fortes et al., 2014), perhaps due to an insufficient amount of protamine to protect DNA or repair DNA breaks. Reduced protamination has been also associated with defects in sperm chromatin structure and correlated with decreased fertility in sires (Dogan et al., 2015).

This phenomenon has also been seen in mice, where disruption of one copy of protamine 1 (*prm1*) or protamine 2 (*prm2*) led to a reduction in protein abundance, an increase in DNA damage, and reduced chromatin compaction (Cho et al., 2003). Interestingly, the disruption of *prm2* did not affect the capacity of the sperm to resume meiosis of MII-arrested eggs, but a decrease in the number of blastocysts was observed, likely due to increased DNA damage affecting embryo development (Cho et al., 2003). Taken together, these findings suggest that protamination-related proteins play a critical role in maintaining DNA integrity in the sperm, which directly impacts early embryonic development (Mateo et al., 2009; Lismer and Kimmins, 2023).

For example, sires with a high sperm DNA fragmentation index (DFI) have a reduced conception rate (32.9%) compared to those with a low DFI (45.3%) (Raval et al., 2024). Furthermore, in sires with high DFI, genes involved in DNA damage response such as *CDK4,* were upregulated, while those associated with DNA repair mechanisms (such as *IL6*, *MLST8*, and *PIK3R3*) were downregulated. The altered expression of key sperm-specific genes may compromise the efficiency of DNA repair pathways, thereby contributing to elevated DNA fragmentation in sperm. Likewise, genes related to the Toll-like receptor signaling pathway and the Jak-STAT pathway, which regulate sperm motility and DNA repair, were downregulated in sperm with high DFI (Raval et al., 2024). These findings highlight the link between sperm DNA fragmentation and subsequent embryo development (Eid et al., 2011). Therefore, selecting sires with a low DNA damage index could be a novel approach to improve sire fertility and enhance reproductive outcomes.

## Impact of proteomic alterations on sperm function and embryo development

Proteins are molecules that modulate various physiological and molecular events in cells, and spermatozoa are not the exception. Alterations in the proteomic profile of sperm can impact its ability to carry out essential biological processes required for fertilization and subsequent embryo development. For example, proteins related to energy production, such as COX7C and proteins involved in the oxidative phosphorylation pathway, which are essential for ATP production and required for sperm motility, have been found in higher abundance in high-fertility (HF) compared to low-fertility (LF) sires (fertility phenotype determined by field fertility). In fact, at the cellular level, HF sires exhibited higher motility compared to LF sires (Gacem et al., 2023).

Proteins associated with locomotion structures, including TPPP2, SSMEM1, and SPAG16, are more abundant in HF sires than LF sires (Gacem et al., 2023). In mice, the disruption of SSMEM1 resulted in a loss of sperm motility and abnormal localization of the Golgi apparatus during spermatid development (Nozawa et al., 2020), which was also linked to alterations in sperm head morphology, abnormal arrangement of cellular organelles, and high incidence of cytoplasmic droplet in the tail.

In humans and mice, in vitro inhibition of TPPP2 results in a reduction in motility and ATP content in sperm cells. This finding was further supported by the experimental knockout of *Tppp2*, which resulted in reduced sperm count and motility (Zhu et al., 2019). Furthermore, the absence of TPP2 resulted in additional molecular events, including the presence of irregular mitochondria, altered expression of electron transport chain molecules, lower ATP levels, reduced mitochondrial membrane potential, and an increase in apoptosis in sperm (Zhu et al., 2019). These findings highlight the critical role of the sperm proteome in regulating key physiological functions essential for male reproductive success. Alterations in the abundance or function of these proteins not only impair sperm motility and structural development but are also closely associated with reduced pregnancy success. In both sperm and seminal plasma, several proteins including BSP-1, BSP-3, BSP-5, spermadhesin-1, ALB, TIMP, AKI, and PEBP1 are found at higher levels in HF sires (sires selected based on daughter pregnancy rates) (Kasimanickam et al., 2019). In contrast, proteins such as CLU, CCT5, CCT8, ELSPbP1, and PSMA6 are more abundant in LF sires (Kasimanickam et al., 2019).

Furthermore, protein localization also influences specific aspects of sperm function relevant to embryo development. For instance, a high overall abundance of BSP proteins in sperm has been associated with acrosome and membrane damage, while the specific localization of BSP5 in the midpiece has been correlated with higher blastocyst rates (Diaz-Miranda et al., 2023). Other proteins involved in sperm motility may also serve as important markers to differentiate HF sires from LF sires. A recent study by Rabaglino et al. (2022) identified 301 differentially abundant proteins and 34 potential biomarker proteins that distinguish HF sires from LF sires (sires classified according to fertility indexes). Notably, the overrepresented biological functions among these proteins were primarily associated with axoneme assembly and sperm motility, reinforcing the critical role of structural and motility-related proteins in determining reproductive success.

It is also possible that groups of proteins (beneficial or detrimental) influence the reproductive success of sires. Aggresomes, which are aggregates of misfolded proteins formed when the protein degradation system is overwhelmed (Corboy et al., 2005), are a good example of detrimental proteins affecting embryo development. A recent study showed that low-performing sires (measured as their ability to produce blastocysts) exhibited a high accumulation of aggresomes, both before and after sperm selection for in vitro fertilization, compared to high-performing sires (Fallon et al., 2023). Further efforts are needed to clarify whether these molecules are produced during spermatogenesis or acquired by sperm as they travel from the epididymis to the reproductive tract. As an example, it has been reported in humans that sperm delivers 11 proteins to the zygote, which are associated with embryo lethality during the early stages of embryonic development (Castillo et al., 2018).

## Oxidative stress in the sperm

Oxidative stress results from an imbalance between the production of reactive oxygen species (ROS) or free radicals and the capacity of cells to neutralize them (Pizzino et al., 2017). In bovine sperm, exposure to high levels of ROS leads to plasma membrane oxidation, alterations in chromatin arrangement, a high incidence of prematurely capacitated sperm, and reduced fertilization and cleavage rates (Silva et al., 2007; Castro et al., 2016). Moreover, high ROS levels negatively affect embryo development, as a decrease in the developmental competence of cleaved embryos to reach the blastocyst stage has been observed (Silva et al., 2007). Interestingly, embryos derived from low-performing sires exhibited higher levels of ROS and autophagy compared to those from high-performing sires (based on their ability to produce embryos) (Fallon et al., 2024).

In sperm, ROS can be produced through two pathways: first, via the nicotinamide adenine dinucleotide phosphate (NADPH) oxidase pathway at the plasma membrane level, and second, through the NADH-dependent oxido-reductase pathway at the mitochondrial level (Agarwal et al., 2003). In any case, if a molecule of O_2_ prematurely binds to a single electron, a superoxide radical (O_2_•−) is produced. This process can continue to accumulate if the electron leakage persists, leading to the formation of additional free radicals, including H_2_O_2_ and NO, which contribute to the generation of ROS and, consequently, oxidative stress (Jena et al., 2021). One of the main consequences of oxidative stress in sperm is the triggering of lipid peroxidation in the plasma membrane, which is enriched with polyunsaturated fatty acids, impairing various sperm traits including acrosome reaction and sperm’s ability to fuse with the oocyte (Wang et al., 2025).

It is well known that physiological levels of ROS are required for the cell to carry a variety of biological functions (Bardaweel et al., 2018; Sosa et al., 2020), but an excessive amount is detrimental. For example, physiological levels of ROS are required for cell growth, proliferation, differentiation, immune response, among other (Hamanaka and Chandel, 2010), but excessive amount of ROS impairs DNA integrity (Rowe et al., 2008). Cells have evolved intrinsic mechanisms to neutralize ROS through both enzymatic and non-enzymatic systems. Although sperm cannot synthesize antioxidants, they rely on an inherited enzymatic system that neutralizes excessive ROS, with semen plasma being a major source of antioxidants. One of the most important enzymes that sperm use to neutralize ROS is superoxide dismutase (SOD) whose antioxidant actions are against O_2_•−.

Seminal SOD activity is positively correlated with overall sperm motility, while reduced SOD activity is associated with a higher DNA fragmentation index (Yan et al., 2014). This data suggests that SOD activity levels are variable and may correlate with the ability of cells to express SOD. In humans, infertile men carrying the *SOD2* rs4880 CC variant, exhibit reduced SOD activity in sperm, affecting sperm concentration and motility (Yan et al., 2014). Likewise, *SOD2* Val16Ala (rs4880) has been linked to male infertility in humans (Ji et al., 2012; Faure et al., 2014). In mice, either complete ablation of *Sod1* (e.g., *Sod1^+/−^*) (Tsunoda et al., 2012) or partial disruption of *Sod1* (e.g., *Sod1^+/−^*) resulted in low levels of SOD1, oligozoospermia, and severely compromised fertilizing ability (Homma et al., 2019).

A recent study in cattle has reported that sexed-sorted semen undergoes a significant loss of natural antioxidants compared to conventional semen (Guo et al., 2023), and a reduction in blastocyst production has been observed when using sexed-sorted semen (Merton et al., 1997; Lu and Seidel, 2004). Moreover, supplementing sexed sorted semen with antioxidants improves embryo development (Li et al., 2018). In humans, lower sperm glutathione peroxidase 1 activity has been correlated with compromised embryo development and morphology on day 5 (Meseguer et al., 2006). Likewise, in goats, supplementing the sperm with glutathione increased the blastocyst production (Zou et al., 2021), indicating that sperm-derived antioxidants could play a critical for subsequent embryo development. All these findings strongly suggest that an adequate intrinsic antioxidant system in sperm is essential for embryo development, and its impairment severely compromises embryo development.

## Centrioles

Centrioles are essential organelles in sperm cells that play critical roles in several biological processes, including spermatogenesis, flagellum formation, centrosome integrity, embryo cytoskeleton organization, fertilization, and embryo development. During spermatogenesis, centrioles are key components of the centrosome, where they modulate the microtubule-organizing center, which is crucial for spindle formation and chromosome segregation, two key events during meiosis. Besides their role in flagellum formation and sperm motility (Avidor-Reiss et al., 2020; Khanal et al., 2021), centrioles play a critical role in embryo development. They contribute to the formation of the zygote’s centrosome, which governs the organization of microtubules, facilitates pronuclear migration, and supports mitotic cell division during early embryonic development.

Therefore, failure in the assembly of the zygotic centrosome can lead to embryo aneuploidy and developmental failure (Avidor-Reiss et al., 2022). Previous research has reported that approximately 65% of zygotes exhibit centrosome anomalies, which could potentially be correlated with embryonic arrest (Chatzimeletiou et al., 2008) and pregnancy loss (Cavazza et al., 2021). In mice, disruption of centrosomes leads to embryonic arrest, and experimental knockout of *Sas-4*, a gene essential for centriole formation, results in embryonic lethality (Bazzi and Anderson, 2014). A recent study in the bovine reported that unexplained subfertility was correlated with abnormal distribution of centriolar biomarkers, such as acetylated tubulin (Turner et al., 2023). Additionally, low-quality sperm from fertile sires (based on pregnancy rates) exhibit an abnormal distribution of centriole proteins (Turner et al., 2023). Interestingly, recent data revealed that embryos derived from HF sires (based on their capacity to produce blastocysts) exhibited an upregulation of genes involved in centriole, centrosome, and spindle formation (Lockhart et al., 2023). Thus, an intact microtubule-organizing machinery for spindle formation and chromosome segregation, and normal zygotic centrosome are essential for successful embryo development.

## Impact of sperm transcriptome on embryo development

The sperm transcriptome may influence several critical aspects of reproductive success. For example, sperm transcripts involved in spermatogenesis (*AFF4* and *BRIP1*), sperm motility (*AK6* and *ATP6V1G3*), capacitation and zona binding (*AGFG1*), and embryo development (*TCF7, MTIF3, EEF1B2,* and *AKIRIN2*) have been shown to be upregulated in HF sires (fertility phenotype determined by conception rate) (Selvaraju et al., 2021). This suggests that there might be transcripts brought by the sperm into the oocyte at the time of fertilization regulating subsequent embryo development. Interestingly, in mice, the knockout of *Mtif3* was found to be embryonic lethal (Rudler et al., 2019), and genetic disruption of *eef1b2* decreased embryo development (Gong et al., 2022). Other transcripts, including *ZNF706*, *CRISP2*, *TNP2*, and *TNP1*, were less abundant in LF sires (based on conception rate), while higher levels of these transcripts were positively associated with conception rate (Prakash et al., 2021). Similar observations were reported by Arangasamy et al. (2011), where the transcriptional abundance of *CRISP2* and *PEBP1* and low abundance of *CCT8* in sperm were positively correlated with SCR. In summary, sperm transcriptome may play a critical role in embryo development and could also serve as a valuable tool for identifying potential molecular markers associated with male fertility.

## Genetic variants for fertility

Although fertility traits generally show low heritability rates (Butler et al., 2019), heritability estimates for certain fertility-related traits such as LH, inhibin, IGF-I, 18-month scrotal circumference, mass activity, progressive motility, and percentage of normal sperm are 0.31, 0.74, 0.44, 0.75, 0.24, 0.15, and 0.25, respectively (Corbet et al., 2013). This is particularly important because genetic selection could serve as a tool to improve sire fertility, thereby enhancing reproductive efficiency, cost-effectiveness, profitability, and genetic progress, among other fertility-related aspects.

For example, single nucleotide polymorphisms in the FSH beta-subunit (*FSHB*) have been associated with lower sperm concentration, compromised acrosome integrity, a significant increase in sperm abnormalities, and lower non-return rates (Dai et al., 2009). In sires, homozygous loss-of-function mutations in *ARMC3*, *CCDC189*, and *QRICH2* result in sperm that are incapable of producing pregnancies in vivo (Pausch and Mapel, 2023). Mutations located on bovine chromosomes BTA 8, BTA 9, BTA 13, BTA 17, and BTA 27 have also been associated with reductions in sire field fertility. This is particularly important, as each of these regions accounts for approximately 5–8% of the variation in sire conception rate, a measure of field fertility (Peñagaricano, 2024). Additionally, mutations on BTA11 and BTA25 may also influence sire fertility, as these regions contain important genes such as *FER1L5*, *CNNM4*, and *DNAH3*, all of which are involved in sperm function (Rezende et al., 2018). Notably, disruption of *Fer1l5* in mice led to sperm incapable of undergoing the acrosome reaction, thereby impairing fertility (Morohoshi et al., 2023). Recent work from our group using high and low-embryo-producing sires, identified mutations in *EGLN1* which is a component of the hypoxia inducible pathway, and *SMG9* a gene involved in mRNA decay to be associated with embryonic cleavage (Davenport et al., 2025 forthcoming). Taken together, while fertility traits in sires exhibit low heritability, recent studies have identified several specific mutations and key genes involved in sperm biology that significantly influence fertilization and embryo development.

## Conclusions

**Figure 1:**
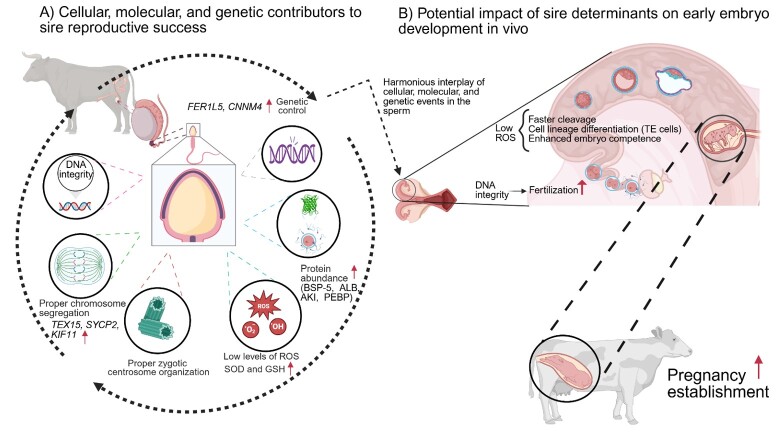
Panel (A) Sire fertility, measured by the ability to produce healthy blastocysts, is influenced by multiple factors at the cellular, molecular, and genetic levels. Among these, sperm DNA integrity is critical for the proper transmission of genetic information necessary for early embryo development. Accurate chromosome segregation is required to ensure that each daughter cell receives the correct set of chromosomes, maintaining genomic stability. Centrioles serve as the foundation for organizing the zygotic centrosome, supporting proper mitotic division. In addition, balanced levels of ROS and antioxidants are essential to activate key signaling pathways required for embryo development. The protein composition of sperm may enhance the embryo’s developmental competence. Furthermore, specific genes can govern a sire’s ability to produce blastocysts and influence overall fertility. Panel (B) A harmonious interplay of cellular, molecular, and genetic events within the sperm modulates its potential to support embryo development and achieve pregnancy success in cattle.

Sire fertility can be influenced by a variety of factors highlighting the potential for developing improved strategies to select sires for enhanced reproductive performance (Figure 1). Traditional morphological evaluation of sperm does not reliably predict a sire's ability to support embryo development. Therefore, selecting sires based on novel traits at the cellular, molecular, and genetic levels represents a promising approach to improving fertility outcomes. Further research aimed at elucidating the differences between LF and HF sires is encouraged, as it will provide valuable insights into which traits can be incorporated into current sire fertility evaluations.

## Data Availability

No research data was used.
